# The novel oncogenic factor TET3 combines with AHR to promote thyroid cancer lymphangiogenesis via the HIF-1α/VEGF signaling pathway

**DOI:** 10.1186/s12935-023-03021-6

**Published:** 2023-09-17

**Authors:** Liyun Yang, Runyu Zhao, Peipei Qiao, Jiaxin Cui, Xiaoping Chen, Jinping Fan, An Hu, Shuixian Huang

**Affiliations:** 1 Department of Otolaryngology Head and Neck Surgery, Gongli Hospital, the Second Military Medical University, Shanghai, 200135 China; 2 Postgraduate Training Base at Shanghai Gongli Hospital, Ningxia Medical University, Shanghai, 200135 China; 3 Department of Otolaryngology Head and Neck Surgery, Changzheng Hospital, the Second Military Medical University, Shanghai, 200135 China

**Keywords:** Thyroid cancer, Lymphangiogenesis, TET3, AHR, HIF-1α/VEGF signaling pathway

## Abstract

**Background:**

Lymphangiogenesis has been reported to play crucial roles in the metastasis of thyroid cancer (THCA), but despite the significant research on lymphangiogenesis in THCA, the precise regulatory mechanism remains unclear.

**Methods:**

Public databases including the Cancer Genome Atlas (TCGA), TIMER, and UALCAN were used to analyze and visualize the expression of TET3 and AHR in THCA, and the correlation between these molecules were used by TIMER. Additionally, RT-PCR and Western Blot were performed to determine the mRNA and protein expression of related proteins. Plate colony formation, wound healing, cell cycle, apoptosis, angiogenesis and transwell assay were used to examine the ability of proliferation, movement, lymphangiogenesis, migration and invasion of THCA cells.

**Results:**

Analysis of the TCGA database revealed higher expression levels of TET3 and AHR in tumor tissue compared to normal tissue in THCA. Additionally, a strong correlation was observed between TET3 and AHR. UALCAN database demonstrated that high expression of TET3 and AHR was associated with advanced THCA TNM stages in THCA patients. Furthermore, TET3 activation accelerated THCA cell proliferation by inducing G2/M phase arrest and suppressing apoptosis, while AHR inactivation reduced THCA cell proliferation by decreasing G2/M phase arrest and promoting apoptosis in vitro. Notably, both TET3 and AHR significantly enhanced THCA cell lymphangiogenesis, migration and invasion. Moreover, TET3 activation and AHR inactivation regulated HIF-1α/VEGF signaling pathway, which ultimately, blocked the HIF-1α/VEGF in THCA cells and impaired their movement, migration and invasion abilities.

**Conclusions:**

The combined action of TET3 and AHR to promote lymphangiogenesis in THCA through the HIF-1α/VEGF signaling pathway, and targeting them might provide a potential treatment strategy for THCA.

## Introduction

Thyroid cancer (THCA) is the fifth most prevalent cancer among women [1], and its incidence tends to rise from adolescence to middle age, with the highest occurrence observed around 55 years for women and 65 years for men. Subsequently, the incidence gradually decreases with advancing ages [2]. Unfortunately, it is projected to become the fourth most common cancer worldwide. In addition, between 1990 and 2013, the age-standardized incidence rate of THCA increased by 20% globally [3]. Despite advancements in treatments such as, immunotherapy, the prognosis for THCA patients remains challenging. The limited understanding of the molecular mechanisms underlying tumor development has posed a significant obstacle to effective treatment. Consequently, exploring the molecular basis of THCA has emerged as a critical research area in recent years.

The growth and metastasis of THCA are closely associated with the development of new lymphatic vessels. The lymphatic network within the tumor serves dual purposes. Firstly, it facilitates the oxygen supply and nutrient metabolism required for tumor cell growth. Secondly, the structural characteristics of lymphatic vessels, such as incomplete tube walls, and varying basement membrane thickness, create favorable conditions for cancer cells to infiltrate the vessels and metastasize [4]. Consequently, lymphangiogenesis plays a crucial role in determining the tumor’s invasive potential and metastatic behavior in THCA. However, the precise underlying mechanism remains unclear.

The HIF-1α/VEGF signaling pathway is a key pathway for the induction of lymphangiogenesis by tumor cells. Tumor growth goes through a lymphangiogeness phase. When the tumor diameter is larger than 2 mm, the tumor cells cannot simply rely on diffuse support. The subunit accumulates in the cytoplasm and is transported to the nucleus, where it binds to the subunit and activates HIF-1α-sensitive target genes. Then, hypoxia in the microenvironment is triggered via the HIF-1α/VEGF pathway, leading to morphological changes in lymphatic endothelial cells, and degradation of the basal membrane at the venous end of the host lymphatic capillary, which stimulate the rapid proliferation of lymphatic endothelial cells. Over time, functional lymphatic capillary loops gradually form and establish connections with the existing lymphatic vessels, thus establishing a complete lymphatic circulation system within the tumor [5].

The aromatic hydrocarbon receptor (AHR) [6] shares a basic HLH/PAS structure with the HIF-1α protein. When AHR binds to the aryl hydrocarbon receptor nuclear translocator (ARNT), the AHR protein and HIF-1α protein form active polymers, which activate the hypoxia response element (HRE) and mediate cellular responses to both toxic chemicals and hypoxic conditions in the microenvironment [7]. TET3 belongs to the DNA demethylation modification enzyme family and is an α-ketoglutarate and Fe^2+^ dependent dioxygenase. It is involved in various cellular processes such as genome imprinting, gene regulation and modulation of repetitive sequences, which play important roles in cancer occurrence and metastasis [8].

In this study, the analysis of public databases, including TCGA, revealed higher expression levels of TET3 and AHR in tumor tissue compared to normal tissue in THCA. Additionally, a strong correlation between TET3 and AHR expression was observed. Further analysis using UALCAN database showed that high expression of TET3 and AHR corelated with TNM stages in THCA patients. Functional experiments demonstrated that activation of TET3 accelerated the proliferation of THCA cells by inducing G2/M phase arrest and suppressing apoptosis. Conversely, the inactivation of AHR reduced THCA cell proliferation by decreasing G2/M phase arrest and inducing apoptosis in vitro. Remarkably, TET3 and AHR were found to significantly promote THCA cell lymphangiogenesis, migration and invasion. Furthermore, TET3 activation and AHR inactivation were shown to regulate HIF-1α/VEGF signaling pathway. Ultimately, blocking HIF-1α/VEGF in THCA cells impaired their ability to move, migrate and invade. Overall, the results indicated that the combined action of TET3 and AHR promotes lymphangiogenesis in THCA through the HIF-1α/VEGF signaling pathway, and targeting TET3 and AHR may hold promise as a potential treatment strategy for THCA.

## Materials and methods

### Public database analysis

Public databases, including the Cancer Genome Atlas (TCGA, https://cancergenome.nih.gov/), TIMER (http://timer.cistrome.org), and UALCAN (http://ualcan.path.uab.edu/analysis.html) were used to analyze and visualize the expression of TET3 and AHR in THCA. In addition, TIMER were used to analyze the correlation between genes.

### Cell culture

The THCA cells (BHT101 and BCPAP cells) were obtained from the Shanghai Institutes for Biological Sciences, Chinese Academy of Sciences. The cells were cultured in DMEM medium, which purchased from YOBIBIO (Shanghai, china) supplemented with 10% FBS, which purchased from YOBIBIO (Shanghai, china) and 1% double antibody was cultured at 37℃ and 5%CO_2_ in a constant temperature and humidity cell incubator. To ensure optimal cell growth, the THCA cells were allowed to reach approximately 80–90% confluency, following which they were detached using trypsin, then centrifuged. The supernatant was discarded, and a fresh medium was added to the cells. Then, the cells were transferred to a new dish for further culturing. For cryopreservation, the cells were harvested, and the resulting cell suspension was centrifuged. The supernatant was removed, and a cryopreservation solution (10%DMSO + 50%FBS + 40% medium) was added to the cell pellet. The cell suspension with cryopreservation solution was carefully transferred to a programmed cryopreservation box for storage.

### siRNA interference

In the experimental group, three siRNAs targeting AHR were designed and synthesized. The day before transfection, cells were seeded into 6-well plates (1.5ml per well) at a density of 5 × 10^5^ cells/ml. The transfection experiments were performed 16 h later, following which the cells were divided into 4 groups. For transfection, each well’s siRNA was mixed with a specific volume of opti-MEM to achieve a final siRNA concentration of 100nM. The total volume of the siRNA mixture was 250 µl and was left at room temperature for 5 min. In a separate tube, 5 µl of lipo2000 transfection reagent was added to 250 µl of opti-MEM and incubated at room temperature for 5 min. The diluted siRNA was then combined with the transfection reagent and incubated at room temperature for 20 min. Finally, 500 µl of the RNA transfection mixture was added to each well containing the cells. After transfection, the culture medium was aspirated 6 h later, and 1.5ml of fresh complete medium was added to each well. Cells were collected 48 h after transfection for quantitative PCR analysis. Effective siRNA sequences were selected based on the PCR results for subsequent functional experiments. The primer sequences used in this study are provided below.

AHR siRNA1, sense (5’-3’): GCUCUGAAUGGCUUUGUAUTT, antisense (5’-3’): AUACAAAGCCAUUCAGAGCCT;

AHR siRNA2, sense (5’-3’): GCCACCAUCCAUACUUGAATT, antisense (5’-3’): UUCAAGUAUGGAUGGUGGCTG;

AHR siRNA3, sense (5’-3’): CCACAUCCACUCUAAGCAATT, antisense (5’-3’): UUGCUUAGAGUGGAUGUGGTA);

NC siRNA, sense (5’-3’): UUCUCCGAACGUGUCACGUdTdT, antisense (5’-3’): ACGUGACACGUUCGGAGAAdTdT.

### Fluorescence quantitative PCR detection

To extract RNA, every 5 × 10^6^ cells were treated with 1ml of Trizol (Invitrogen). The mixture was thoroughly mixed by pipetting and left at room temperature for 5 min. Next, 0.2ml of chloroform was added to each 1ml of Trizol solution. The mixture was vigorously shaken for 15 s and left at room temperature for 2–3 min. Following this, the solution was centrifuged at 4℃ for 15 min at 12,000 g. From the resulting upper colorless aqueous phase, 0.3ml was carefully transferred to a separate EP tube, taking care not to aspirate the lower phase. To the transferred aqueous phase, 0.3ml of isopropyl alcohol was added, mixed thoroughly, and left at room temperature for 10 min. The solution was then centrifuged at 4℃ for 15 min at 12,000 g. The supernatant was discarded, and the RNA pellet was washed with 1ml of pre-cooled 75% ethanol by carefully inverting and mixing several times. After centrifugation at 4℃ and 12,000 g for 5 min, the supernatant was removed, and the RNA pellet was air-dried at room temperature for 5–10 min. Finally, the RNA pellet was dissolved in 20–50µL of DEPC-treated water, and its concentration and purity were measured using a nucleic acid detector (1.8 < A260/A280 < 2.0). The RNA was stored on ice or at -80℃ for a long time until use.

For quantitative PCR, the reaction system was prepared according to the instructions provided with the kit. Briefly, the total reaction mixture was prepared, thoroughly mixed, and distributed onto a 96-well plate. Then, the cDNA solution was added to each well, and the plate was sealed with a membrane and centrifuged for machine-based detection.

### Plate colony formation assay

The cells were seeded onto 6-well plates at a density of 1 × 10^3^ and 2 × 10^3^ cells/well, cultured in DMEM with 10% FBS for 3 weeks, then stained with crystal violet for 30 min. Then, the cell colonies in every well were counted.

### Angiogenesis experiment

Here, 50 µl of Matrigel (10 mg/ml) was added into each well of a 96-well plate and placed in an incubator at 37℃ for about 30 min. After coagulation, the cells were inoculated, then the cells from each group were digested and collected. Further, each well was inoculated with 20,000 cells and cultured for 8 h, following which images were taken for analysis.

### Apoptosis detection

All medium and cell suspension digested by trypsin were collected and centrifuged at 1000 rpm for 5 min. Then, the medium was removed, and after centrifugation, the cell precipitates were suspended in 100µL 1×Binding buffer and transferred to the flow tube. Next, 5µL FITC-Annexin V and 10µL PI were added into the flow tube. One tube of non-stained cells was prepared as flow control. The contents were gently mixed and incubated at room temperature away from light for 15 min. In addition, 400µL binding buffer was added and mixed into each tube, and the tubes were detected by flow cytometry within 1 h.

### Cell cycle detection

The cells were centrifuged and washed once with PBS to remove the supernatant. Then, 1ml of 70% ethanol pre-cooled at -20℃ was added to the cell precipitate and fixed overnight at -20℃. After centrifuging at 400 g for 5 min, 1ml of PBS was added for another round of centrifugation. Next, the cells were treated with 200µL of staining buffer, 2µL of RNase (10 mg/mL), and 10µL of PI (1 mg/mL) and incubated at 37℃ for 30 min in the light. Finally, flow cytometry was used to detect the cells.

### Wound healing

For this experiment, 1 × 10^6^ cells were inoculated into 6-well plates. After overnight incubation, the cells were scratched using a 10 µl pipette tip and photographed using a powered microscope with a 4x or 10x magnification.

### Cell transwell assay

Invasion experiments were conducted using Matrigel from Becton Dickinson Labware (Bedford, MA, USA). In each Transwell (Boyden Transwell chambers, Corning, MA, USA), 5 × 10^4^ cells were inoculated in 100µL of serum-free medium cell suspension, while a complete medium was added to the lower chamber. Following 24 h of culture, the cells were stained with paraformaldehyde and crystal violet, then wiped with cotton swabs, washed with PBS, and allowed to dry at room temperature. Three fields were randomly selected for each chamber to capture photos under a light microscope and count the cells.

### Detection of protein expression by western blot

For each sample, 1 × 10^7^ cells were combined with 1ml of RIPA lysate (Thermo Scientific) and protease inhibitor, homogenized and placed on ice for 10 min, centrifuged at 14,000 g and 4℃ for 10 min, following which the supernatant was collected, and protein concentration was detected by the BCA method. An equal amount of protein was combined with 5×SDS-PAGE Loading Buffer (Thermo Scientific) and thoroughly mixed. The concentration of the acrylamide separation gel used was 10%. The supernatant, obtained after the aforementioned heating treatment, was added to each sample well for electrophoresis (70 V/30min, 120 V/60min). Wet transfer was performed at a constant current of 250mA for 90 min. The membrane was then blocked with 5% skim milk powder at room temperature for 1 h. Each antibody was diluted in 5% skim milk powder at a ratio of 1:1000 and incubated overnight at 4℃. The primary antibody was subsequently removed, and the membrane was washed thrice with TBST for 5 min each time. Next, an HRP-labeled secondary antibody (1:5000) was added, and the membrane was incubated at room temperature for 1 h. After incubating with the secondary antibody, the membrane was washed thrice with TBST for 5 min each. Finally, the membrane was developed using ELC luminescent solution (chemiscope6100) for imaging.

### Statistical analysis of data

The data are presented as mean ± SD. Statistical analysis was performed using GraphPad Prism (GraphPad Software, San Diego, CA) software (T-test). A p-value of less than 0.05 was considered to indicate a significant statistical difference.

## Results

### The expressions of TET3 and AHR in pan-cancer

**Fig. 1:**
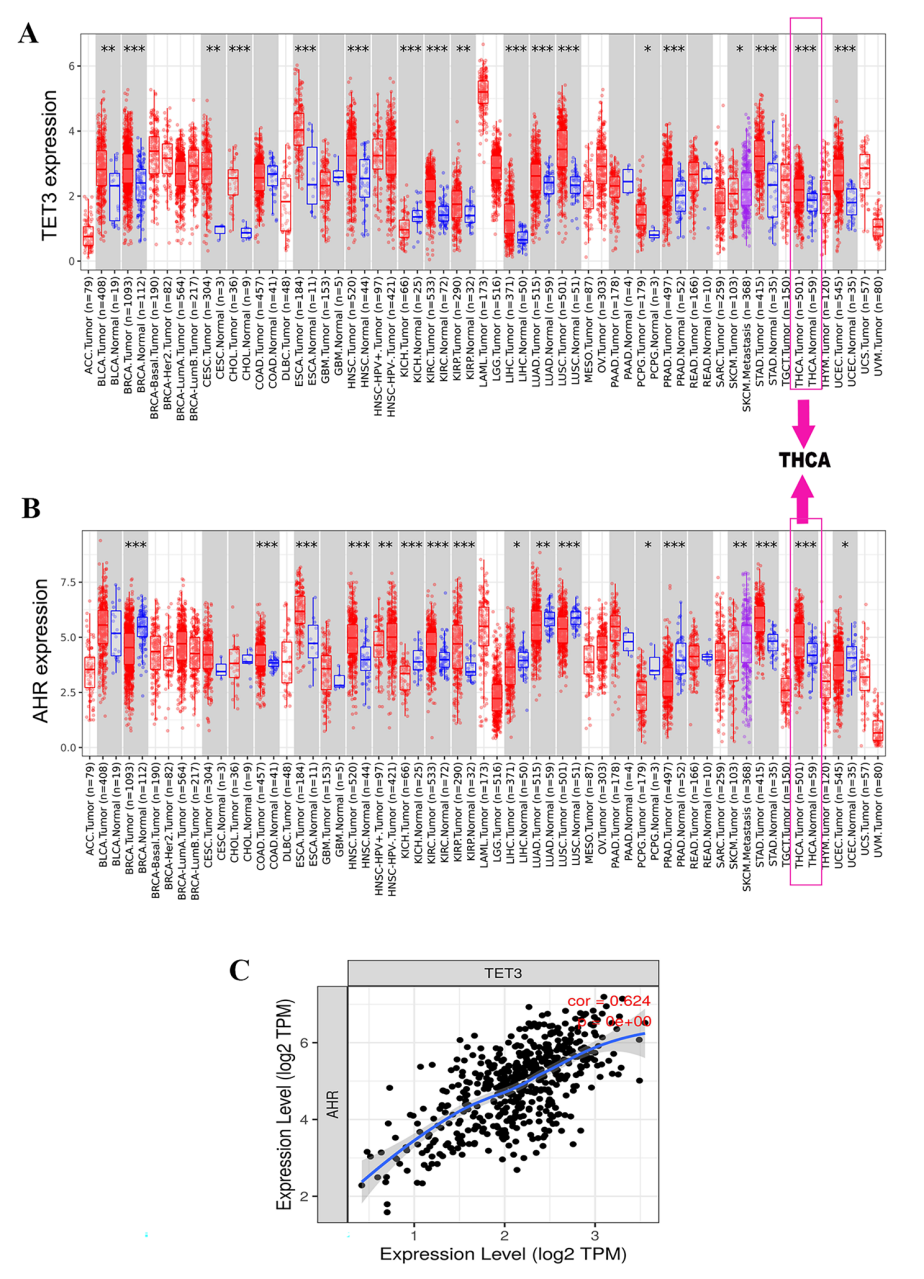
TET3 and AHR expressions are higher in THCA tissues, and TET3 is positively correlated with AHR. **AB** Differences in TET3 and AHR mRNA expression levels between TCGA-THCA tissues and adjacent TCGA-THCA normal tissues. The distributions of gene expression levels are displayed using box plots. **C** Correlations between the mRNA expression level of TET3 and AHR in THCA were determined in the TIMER database, respectively

To investigate the expression of TET3 and AHR in cancer, we conducted an analysis using the TCGA database. Our findings revealed significantly higher expression levels of TET3 and AHR in TCGA-THCA tissues compared to adjacent TCGA-THCA normal tissues (p < 0.001, Fig. 1A and B). Additionally, we examined the correlation between the mRNA expression levels of TET3 and AHR in THCA using the TIMER database, and a strong correlation (Pearson correlation coefficient ≈ 0.62, p < 0.001) was observed between TET3 and AHR (Fig. 1C).

### The high expression of TET3 and AHR were correlated with THCA TNM stages of THCA patients

**Fig. 2:**
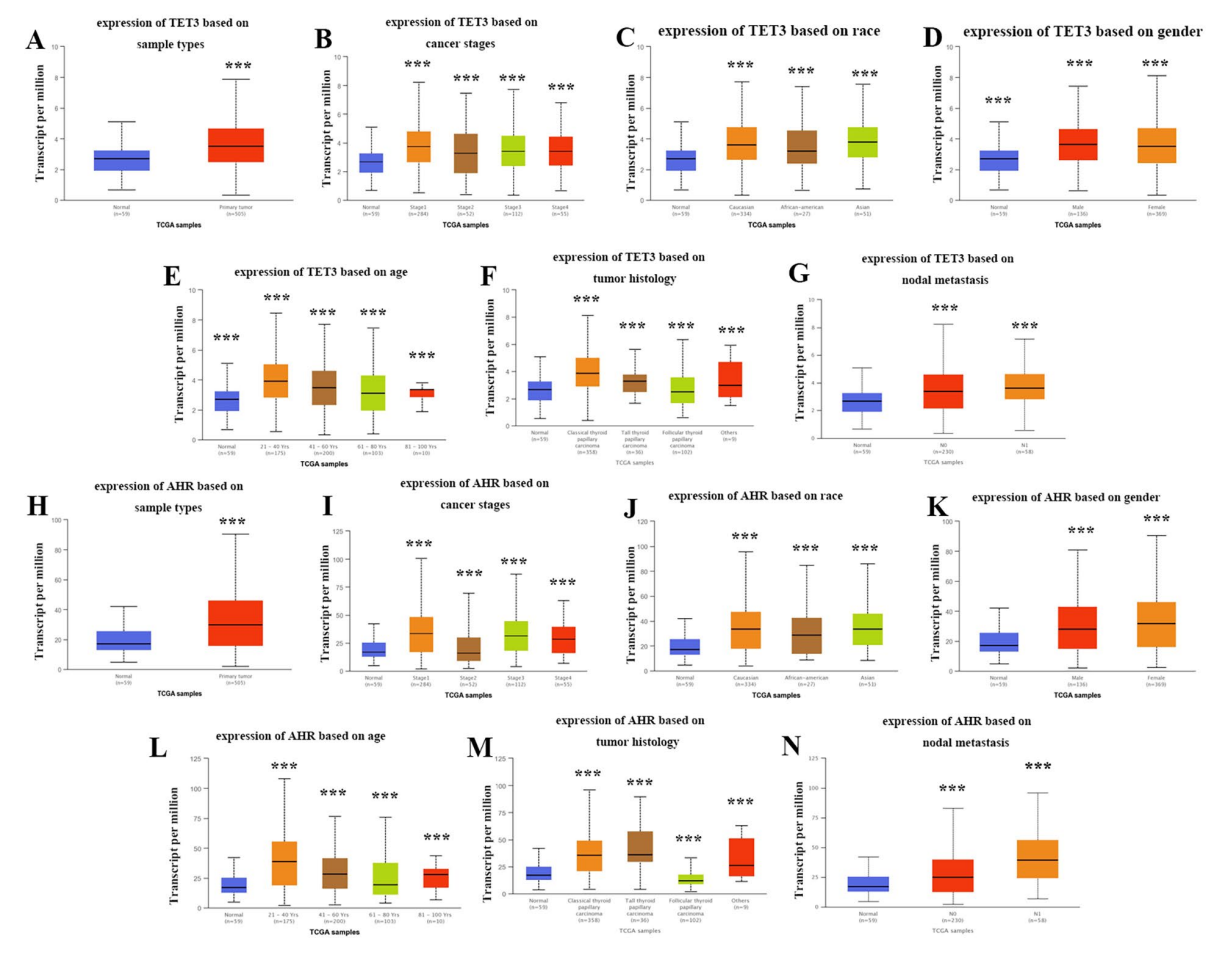
Relationship between the expressions of TET3 and AHR mRNA and the prognosis of THCA patients. **A.H.** The expressions of TET3 and AHR in tumor tissue were significantly higher than in normal tissue in the THCA data set. **B.I.** The TET3 and AHR expression levels were significantly lower in the tumor grade 1 subgroup than in the tumor grade 2/3 subgroup and lower in the tumor grade 2 subgroup than in the tumor grade 3 subgroup. **C.J** The expressions of TET3 and AHR were higher in Caucasian, African-Americans and Asians. **D.K.** The expressions of TET3 and AHR were higher in women. **E.L.** The expressions of TET3 and AHR were higher in patients aged 21–60 years. **F.M.** The expressions of TET3 and AHR were classical and tall THCA. **G.N.** Patients with lymph node metastasis demonstrated a higher expression of TET3 and AHR.

Analysis of the UALCAN database further supported our findings on the expression of TET3 and AHR in THCA tissues. We observed significantly higher expression levels of TET3 and AHR in THCA tissues compared to adjacent normal tissues (Fig. 2A H). Within the THCA patient subgroup, TET3 and AHR expression levels were significantly lower in the tumor normal subgroup compared to the tumor grade 1/2/3/4 subgroups. Additionally, TET3 and AHR expression levels were lower in the tumor grade 1 subgroup compared to the tumor grade 2/3 subgroups and lower in the tumor grade 2 subgroup compared to the tumor grade 3 subgroup (P < 0.001, Fig. 2B and I). We also observed higher expression levels of TET3 and AHR in different racial groups, including Caucasian, African-American, and Asian populations (P < 0.001, Fig. 2C J). Moreover, TET3 and AHR expression levels were higher in female patients (P < 0.001, Fig. 2D K). Furthermore, increased expression of TET3 and AHR was observed in the age group of 21–60 years (P < 0.001, Fig. 2E L), as well as in classical and tall THCA subtypes (P < 0.001, Fig. 2F M). UALCAN database analyses based on the THCA dataset indicated that patients with lymph node metastasis exhibited higher expressions of TET3 and AHR (P < 0.001, Fig. 2G N).

### TET3 and AHR promotes the growth of THCA cells by inducing G2/M phase arrest and restraining apoptosis in vitro of THCA cells

**Fig. 3:**
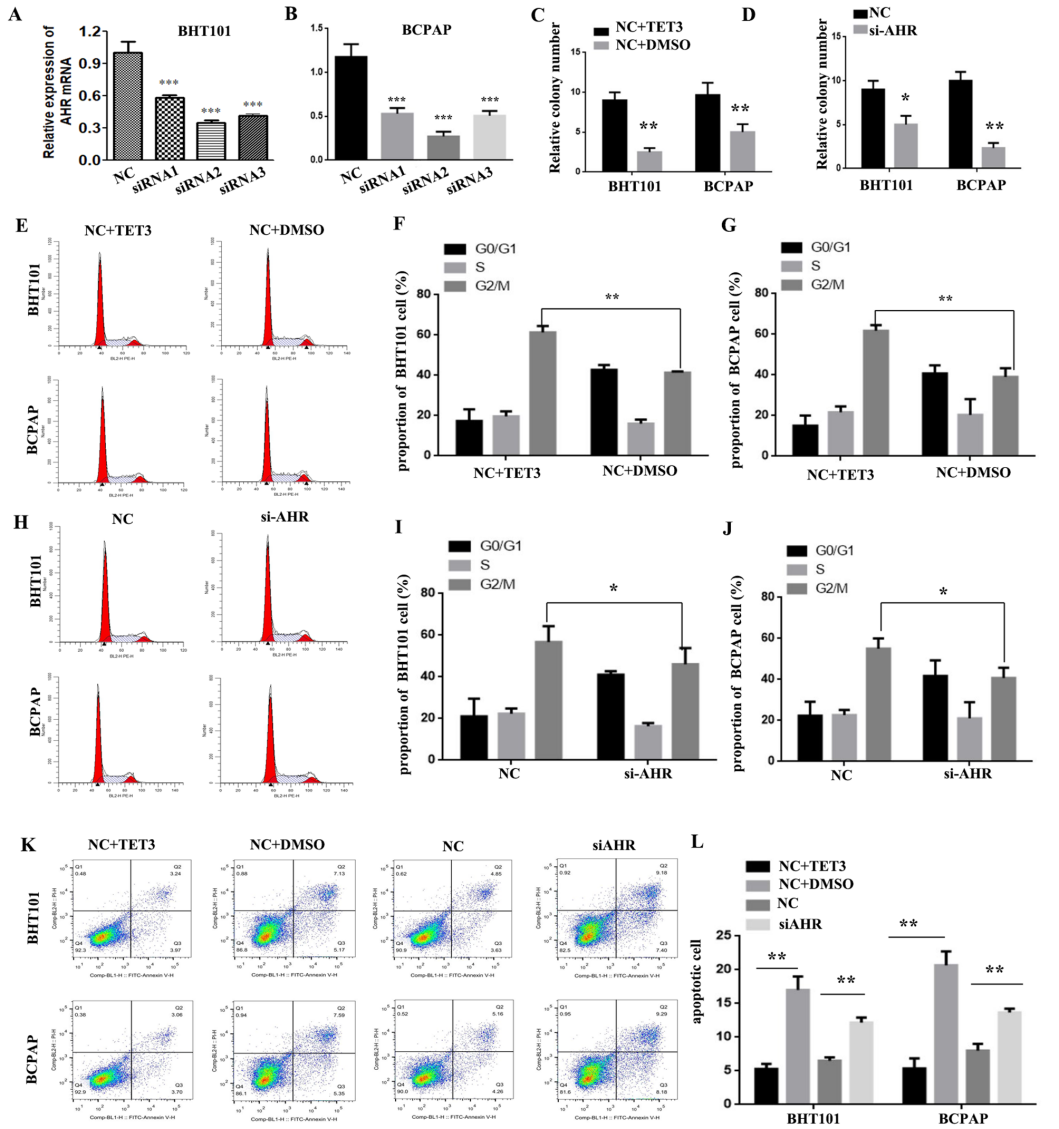
TET3 and AHR promote the growth of THCA cells by inducing G2/M phase arrest and restraining apoptosis in vitro. **A.B.** RT-PCR of AHR expression in BTH101 and BCPAP cells with the transient transfection of AHR siRNA (siRNA1, siRNA2 and siRNA3). **CD** TET3 and AHR accelerated the proliferation of THCA cells in colony formation assay. **EFGHIJ** The TET3 and AHR activation induced THCA cells G2/M phase arrest. **KL.** TET3 and AHR activation increased the percentage of THCA cells in the G2/M phase

Given the crosstalk between the TET3 and AHR overexpression and THCA proliferation, we further elucidated the biological functions of TET3 and AHR in THCA using plate colony formation assay, and the cellular period and apoptosis were tested by flow cytometry. TET3 expression was found to be upregulated following the addition of TET3 small molecules. The expression of AHR was then silenced by transfecting AHR-siRNA (siRNA1, siRNA2 and siRNA3) in BHT101 and BCPAP cells (**P < 0.01, Fig. 3A and B). All three siRNAs significantly reduced the expression of AHR, with siRNA2 exhibiting the most effective silencing; therefore, it was chosen for subsequent experiments. Plate colony formation assay results showed enhanced proliferation in BHT101/(NC + TET3), BCPAP/(NC + TET3), BHT101/NC, and BCPAP/NC cells compared to BHT101/(NC + DMSO), BCPAP/(NC + DMSO), BHT101/si-AHR, and BCPAP/si-AHR cells (*P < 0.05, **P < 0.01, Fig. 3A and B). Furthermore, TET3 activation induced G2/M phase arrest in THCA cells, while AHR inactivation reduced G2/M phase arrest (*P < 0.05, **P < 0.01, Fig. 3E J). Additionally, TET3 activation decreased cell apoptosis, whereas AHR inactivation increased cell apoptosis (**P < 0.01, Fig. 3K L). These findings suggest that TET3 and AHR promote the growth of THCA cells by inducing G2/M phase arrest and suppressing apoptosis in vitro.

### TET3 and AHR promote the lymphangiogenesis, migration and invasion of THCA cells

**Fig. 4:**
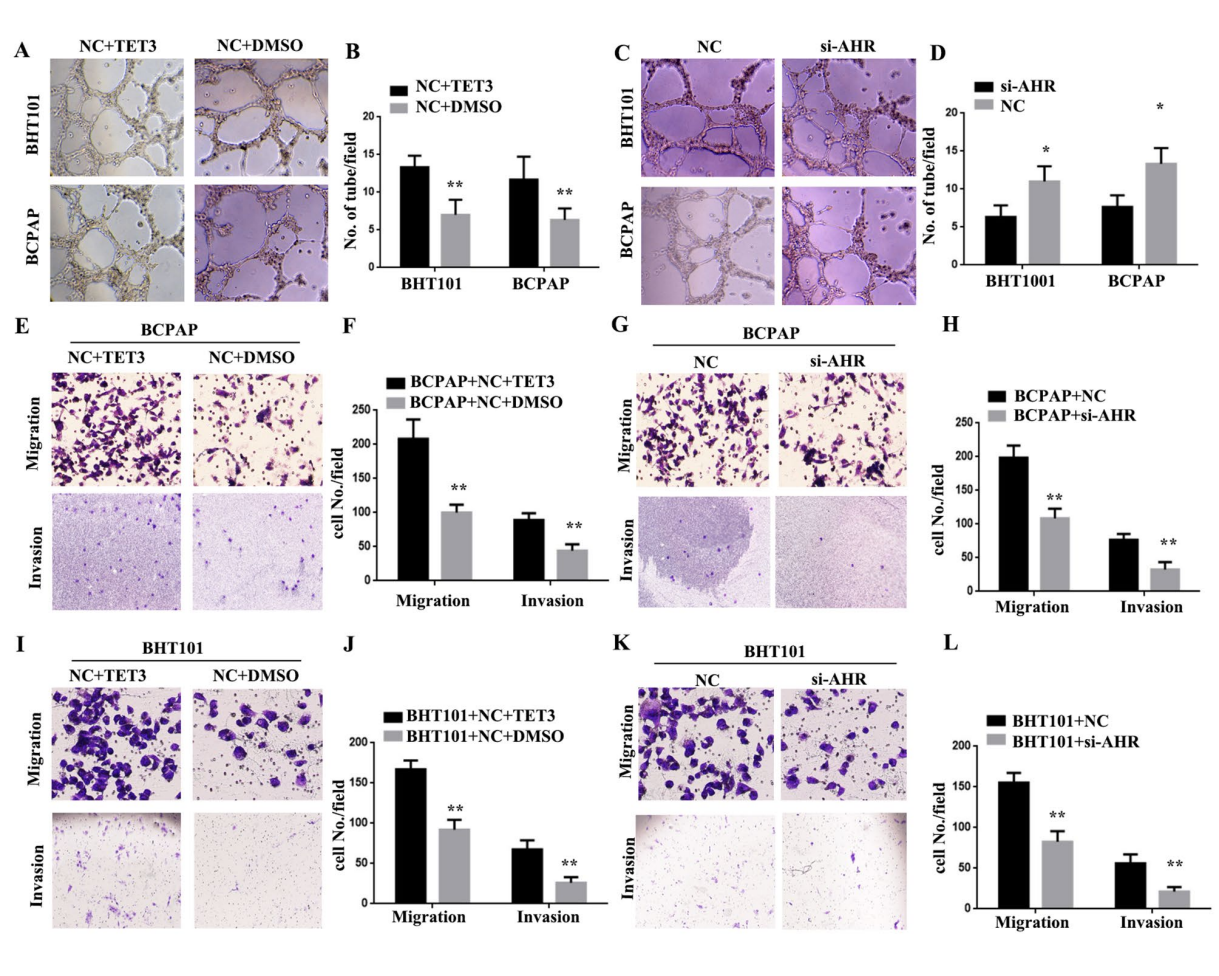
TET3 and AHR promote lymphangiogenesis, migration and invasion of THCA cells. **A.C.** Vasculogenic effects assessed based on capillary-like tubule structure formation. **B.D.** Number of tubes in every field (*P < 0.05, **P < 0.01). **E.G.** Transwell invasion assays performed to assess the migration and invasion abilities of THCA cells. **F.H.** Cell numbers in every field (*P < 0.05, **P < 0.01)

To further investigate the role of TET3 and AHR in the metastasis of THCA, we examined their effects on THCA lymphangiogenesis, migration, and invasion. The vasculogenic effects were assessed by evaluating capillary-like tubule structure formation. Tubule formation was significantly increased in BHT101/(NC + TET3) and BCPAP/(NC + TET3) cells (***P < 0.01, Fig. 4A and B). Furthermore, the results of the angiogenesis experiment demonstrated a significant decrease in tubule formation in BHT101/si-AHR and BCPAP/si-AHR cells (*P < 0.05, Fig. 4 C and 4D). In addition, we performed transwell assays to assess the migration and invasion properties of THCA cells. Upregulation of TET3 markedly enhanced the ability of migration and invasion in BHT101 and BCPAP cells (**P < 0.01, Fig. 4E F, 4I, and 4 J). Conversely, the knockdown of AHR significantly decreased the ability of migration and invasion in BHT101 and BCPAP cells (**P < 0.01, Fig. 4G H, 4 K, and 4 L). These findings indicate that TET3 and AHR promote lymphangiogenesis, migration, and invasion of THCA cells.

### TET3 combined AHR to promote THCA lymphangiogenesis by HIF-1α/VEGF signaling pathway

**Fig. 5:**
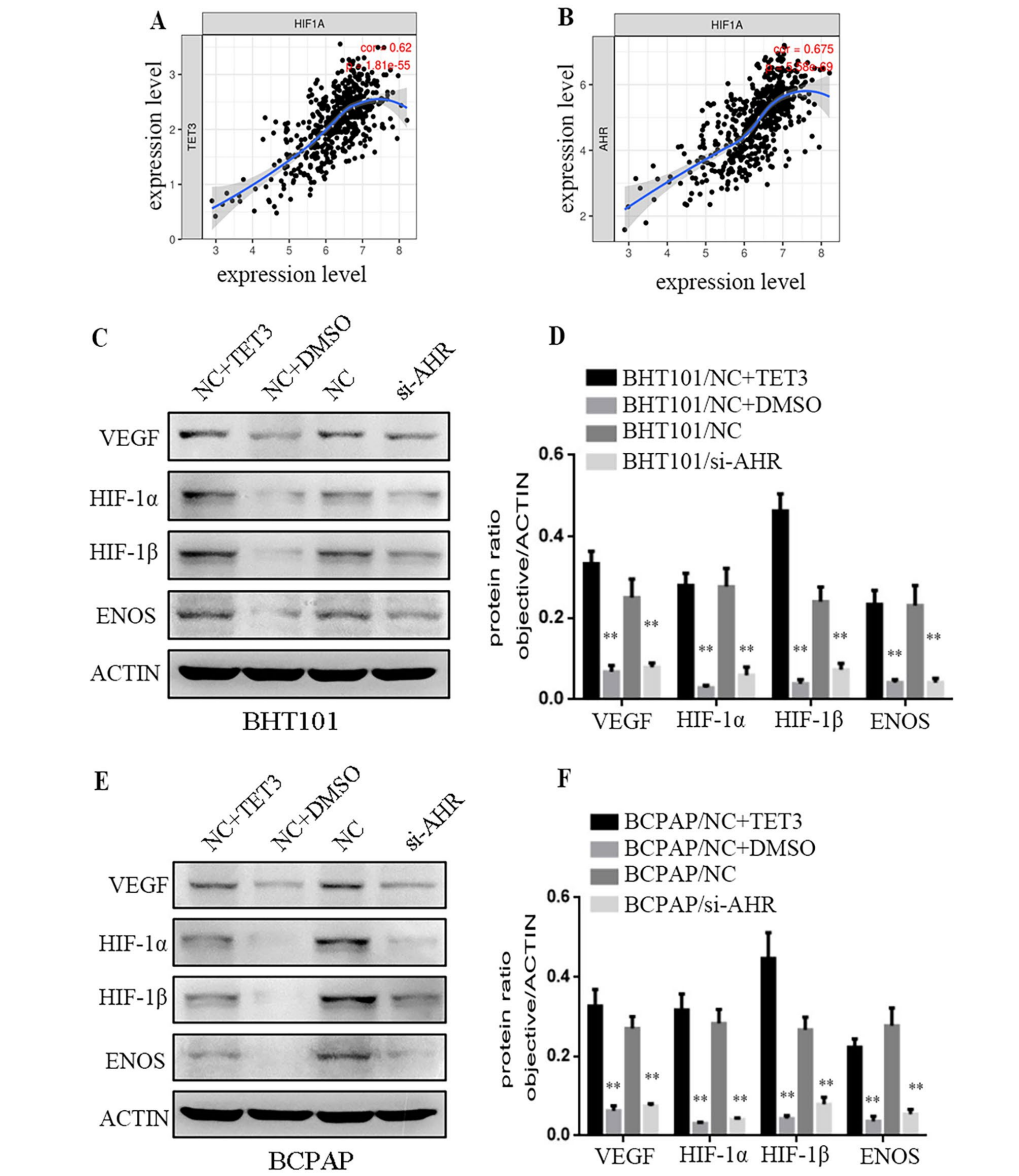
HIF-1α/VEGF signaling pathway plays an important role in the metastasis of THCA. **A.B.** The correlations between TET3 and AHR and key genes of HIF-1α/VEGF signaling pathway in THCA in the TIMER database.**C.** Levels of AHR, VEGF, HIF-1α, HIF1β and ENOS were increased in BHT101/(NC + TET3) and BCPAP/(NC + TET3) cells. **D.** Protein ratio in cells (**P < 0.01). **E.** Levels of AHR, VEGF, HIF-1α, HIF1β and ENOS were increased in BHT101/NC and BCPAP/NC cells. **F.** Protein ratio in cells (**P < 0.01)

The HIF-1α/VEGF signaling pathway has been recognized to play a crucial role in cancer metastasis [9–12]. To explore the potential involvement of TET3 and AHR in this pathway, we analyzed the correlations between TET3, AHR, and key genes of the HIF-1α signaling pathway using the “correlation” module of the TIMER database. Our analysis revealed significant positive correlations between TET3, AHR and HIF-1α (Fig. 5A and B) and suggested that TET3 and AHR may play a role in mediating the HIF-1α signaling pathway to promote THCA metastasis. In addition, our present researches indicated VEGF signaling pathway played a crucial role in head and neck squamous cell carcinoma [13, 14]. To further demonstrate the importance of HIF-1α and VEGF in THCA, we measured the protein expressions using Western Blot. The protein levels of AHR, VEGF, HIF-1α, HIF-1β, and ENOS were increased in BHT101/(NC + TET3) and BCPAP/(NC + TET3) cells. Conversely, the protein levels of AHR, VEGF, HIF-1α, HIF-1β and ENOS were decreased in BHT101/si-AHR and BCPAP/si-AHR cells (**P < 0.01, Fig. 5 C-5 F). These findings indicate that TET3 and AHR, in combination, promote THCA lymphangiogenesis through the HIF-1α/VEGF signaling pathway.

### Blocking HIF-1α impairs the movement, migration and invasion of THCA cells

**Fig. 6:**
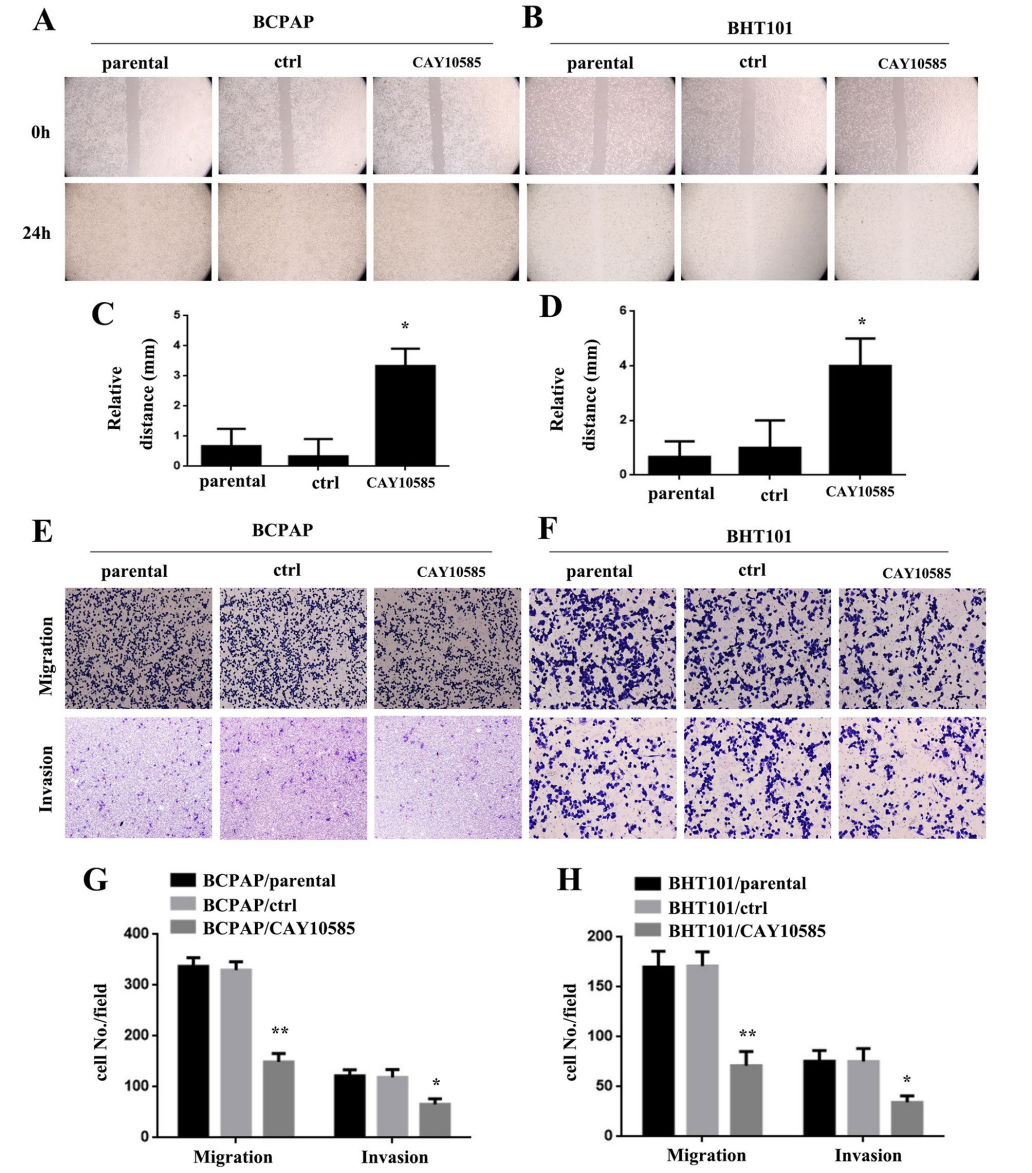
Blocking HIF-1α impairs THCA cells’ movements, migration and invasion abilities. **AB** BCPAP/CAY10585 and BHT101/CAY10585 cells were less motile at 24 h compared with BCPAP/parental and BCPAP/ctrl, and BHT101/parental and BHT101/ctrl cells. **C.D.** Wound size in BCPAP/CAY10585 and BHT101/CAY10585, BCPAP/parental and BCPAP/ctrl, and BHT101/parental and BHT101/ctrl cells (*P < 0.05). **E.F.** BCPAP/CAY10585 and BHT101/CAY10585 cells exhibited reduced movement through Matrigel compared to BCPAP/parental and BCPAP/ctrl, as well as BHT101/parental and BHT101/ctrl cells. **G.H.** Cell number in every field in BCPAP/CAY10585 and BHT101/CAY10585, BCPAP/parental and BCPAP/ctrl, and BHT101/parental and BHT101/ctrl cells (*P < 0.05, **P < 0.01)

To further investigate the potential of inhibiting the tumor-promoting effects of THCA by blocking HIF-1α expression, we used the HIF-1α inhibitor CAY10585 in BCPAP and BHT101 cells. In the wound-healing assay, BCPAP/CAY10585 and BHT101/CAY10585 cells showed reduced motility at 24 h compared to BCPAP/parental, BCPAP/ctrl, BHT101/parental, and BHT101/ctrl cells (*P < 0.05, Fig. 6A and D). Moreover, the results of the migration and invasion assay demonstrated that BCPAP/CAY10585 and BHT101/CAY10585 cells exhibited decreased movement through Matrigel compared to BCPAP/parental, BCPAP/ctrl, BHT101/parental, and BHT101/ctrl cells (*P < 0.05, **P < 0.01, Fig. 6E H).

### Blocking VEGF impairs the movement, migration and invasion of THCA cells

**Fig. 7:**
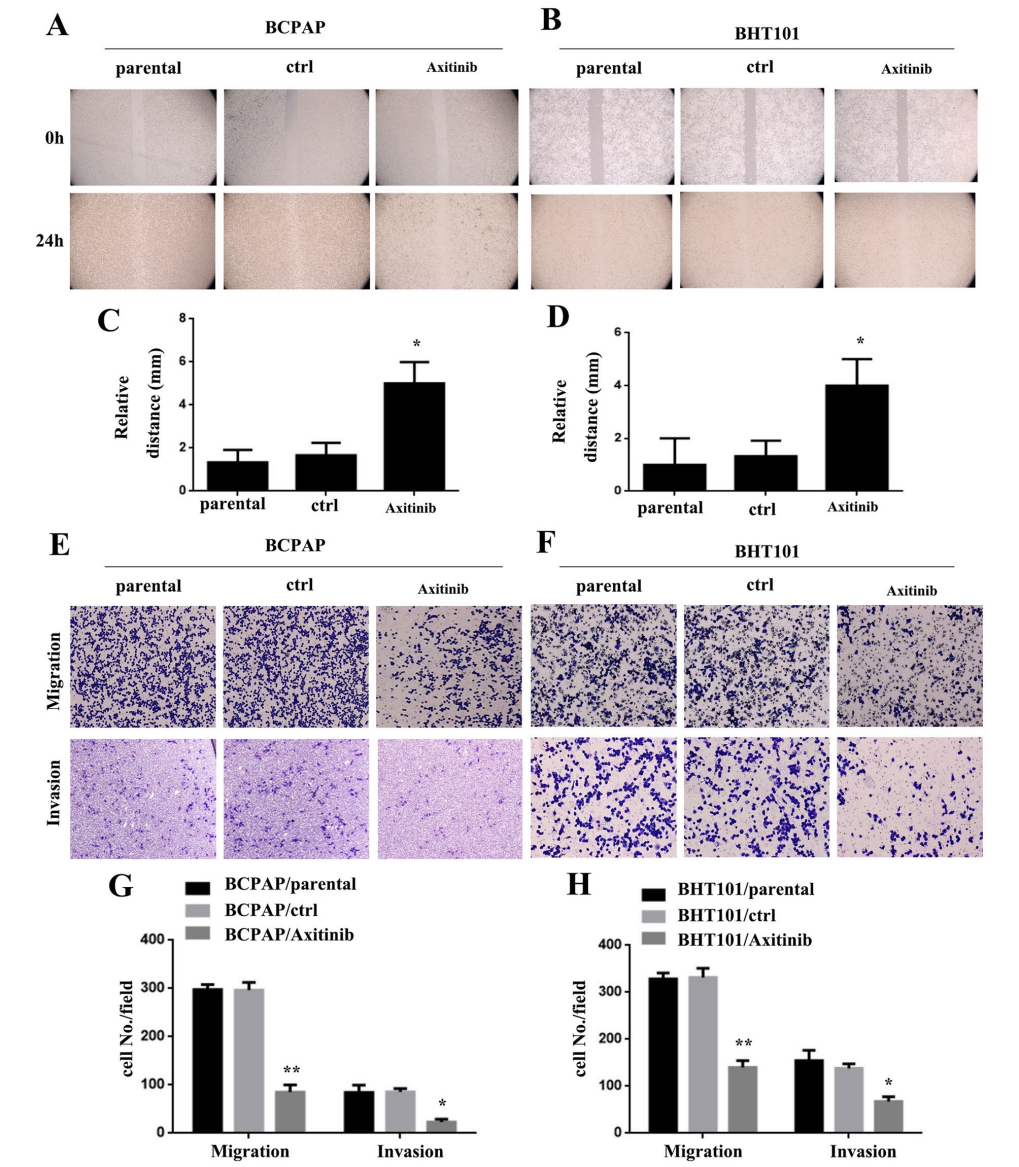
Blocking VEGF impairs THCA cells’ movement, migration and invasion abilities. **AB** BCPAP/Axitinib and BHT101/Axitinib cells were less motile at 24 h compared with BCPAP/parental and BCPAP/ctrl, and BHT101/parental and BHT101/ctrl cells. **CD** Wound size of BCPAP/Axitinib and BHT101/Axitinib, BCPAP/parental and BCPAP/ctrl, and BHT101/parental and BHT101/ctrl cells (*P < 0.05). **EF** BCPAP/ Axitinib and BHT101/Axitinib cells exhibited reduced movement through Matrigel than BCPAP/parental and BCPAP/ctrl, and BHT101/parental and BHT101/ctrl cells. **GH** Cell number in every field in BCPAP/Axitinib and BHT101/Axitinib, BCPAP/parental and BCPAP/ctrl, and BHT101/parental and BHT101/ctrl cells (*P < 0.05, **P < 0.01)

To further investigate the potential of inhibiting the tumor-promoting effects of THCA by blocking VEGF expression, we used the VEGF inhibitor Axitinib in BCPAP and BHT101 cells. In the wound-healing assay, BCPAP/Axitinib and BHT101/Axitinib cells showed reduced motility at 24 h compared to BCPAP/parental, BCPAP/ctrl, BHT101/parental, and BHT101/ctrl cells (*P < 0.05, Fig. 7A and D). Moreover, the results of the migration and invasion assay demonstrated that BCPAP/Axitinib and BHT101/Axitinib cells exhibited decreased movement through Matrigel compared to BCPAP/parental, BCPAP/ctrl, BHT101/parental, and BHT101/ctrl cells (*P < 0.05, **P < 0.01, Fig. 7E H).

## Discussion

Tumor metastasis is the leading cause of cancer-related deaths globally, in which lymphangiogenesis and lymph node metastasis have been found to play a significant role [15]. However, the precise molecular and cellular regulators involved in lymphangiogenesis remain poorly understood.

This present study was designed to investigate the biological role of TET3 and AHR, as well as explore their underlying mechanisms in THCA. Through bioinformatics analysis, we observed a strong correlation between high expressions of TET3 and AHR with THCA tissues of THCA patients. Furthermore, our data revealed a potential molecular mechanism in which the oncogenic factor TET3 interacts with AHR to promote THCA lymphangiogenesis by activating the HIF-1α/VEGF signaling pathway. These findings collectively highlight the critical role of the oncogenic factor TET3 in THCA metastasis and provide novel insights into the molecular mechanisms driving THCA metastasis.

Previous studies have indicated the potential significance of TET3 in tumor recurrence and metastasis in various types of cancer, including ovarian [16], endometrial [17], breast [18], pancreatic [19], and thyroid cancer (THCA) [20]. Accumulating evidence suggests that TET3 plays a crucial role in cell cycle regulation, anti-apoptosis, therapy resistance, and epithelial-mesenchymal transition (EMT) [21, 22].

Previous studies reported that TET3 and AHR have multifunctional roles in tumor cell proliferation and invasion. TET3 upregulation has been shown to significantly promote THCA growth and metastasis, while silencing the expression of TET3 inhibits THCA growth and metastasis. Similarly, AHR has also been shown to play a crucial role in tumor cell proliferation and invasion. The activation of TET3 was shown to accelerate THCA cell proliferation by inducing G2/M phase arrest and suppressing apoptosis, whereas the inactivation of AHR reduced THCA cell proliferation by decreasing G2/M phase arrest and promoting apoptosis in vitro. Taken together, the overexpression of TET3 and AHR in THCA indicates their involvement in promoting tumor aggressiveness and targeting them could potentially serve as a treatment strategy for THCA.

Several signaling pathways, such as PI3K [23], FAK [24], NF-κB [25], JAK/STAT3 [26] and the HIF-1α/VEGF signaling pathway [27], have been implicated in the process of lymphangiogenesis during cancer metastasis, among which the HIF-1α/VEGF signaling pathway is considered one of the key pathways associated with lymphangiogenesis and metastasis.

TET3 is a member of the DNA demethylation modifying enzyme family and has been reported to be involved in gene regulation, repression of repetitive sequences and cancer development. AHR, upon ligand binding, can activate multiple signaling pathways [8]. Studies on prostate cancer have confirmed that the interaction between TET3 and AHR can influence the expression of various factors. It has been observed that TET3-AHR interaction leads to decreased phosphorylation of AKT, an HIF-1α pathway-related factor, while promoting the phosphorylation of β-catenin and its downstream target genes cyclin D1 and c-myc [28]. Some scholars believe that TET3 can reduce CYP1A1 expression when functioning as a ligand of AHR, which in turn affects nuclear transport and the binding of AHR to DNA [29]. In studies performed on inflammatory diseases, TET3 has been reported to regulate T cell differentiation after binding with AHR [30]. However, there have been few studies on the effects of the TET3-AHR interaction on tumor lymphangiogenesis.

In THCA, TET3 has been identified as a promoter of lymphangiogenesis through multiple signaling pathways, and its expression has been associated with the upregulation of multiple factors involved in lymphangiogenesis, such as VEGF and eNOS. Based on these findings, we conducted a preliminary study to investigate the combined effect of TET3 and AHR on lymphangiogenesis in THCA. Using WT and AHR^−/−^ tumor cell models, we observed that TET3 could bind to AHR, and this binding can be disrupted by AHR-specific siRNA.

Our study reveals a novel mechanism by which TET3 downregulates the HIF-1α signaling pathway via AHR, leading to the promotion of lymphangiogenesis in THCA. To our knowledge, there have been no previous reports on the regulation of the HIF-1α signaling pathway by TET3 and its molecular mechanism in promoting lymphangiogenesis in THCA. Therefore, our findings provide valuable insights into the molecular mechanism of TET3 in lymphangiogenesis in THCA, which may hold significant potential for the prevention and treatment of THCA.

In conclusion, our findings indicate that the combined action of TET3 and AHR promotes lymphangiogenesis in THCA through the HIF-1α/VEGF signaling pathway. Therefore, targeting TET3 and AHR could serve as an effective treatment strategy for THCA.

## Data Availability

The data and material from the current study can be made available from the corresponding author upon reasonable request.
